# Inactivated Type ‘O’ Foot and Mouth Disease Virus Encapsulated in Chitosan Nanoparticles Induced Protective Immune Response in Guinea Pigs

**DOI:** 10.3390/ani15243540

**Published:** 2025-12-09

**Authors:** Kalaivanan Ramya, Subodh Kishore, Palanisamy Sankar, Ganesh Kondabatulla, Bedaso Mamo Edao, Ramasamy Saravanan, Kumaraguruban Karthik

**Affiliations:** 1 Indian Veterinary Research Institute, Hebbal, Bangalore 560024, India; subodhkishore@gmail.com (S.K.); kondabattula@gmail.com (G.K.); bedasomamo@yahoo.com (B.M.E.); 2 Veterinary College and Research Institute, Tamil Nadu Veterinary and Animal Sciences University, Udumalpet 642205, India; drpsankarster@gmail.com (P.S.); karthik_2bvsc@yahoo.co.in (K.K.); 3 Veterinary College and Research Institute, Tamil Nadu Veterinary and Animal Sciences University, Namakkal 637002, India

**Keywords:** FMDV, chitosan, nanoparticles, immune response, protection, guinea pig, route of immunization

## Abstract

Foot and mouth disease is a contagious viral disease infecting ungulates, with great economic impact on farmers’ income; it is primarily controlled using inactivated vaccines, which have certain limitations, such as short-lived immunity and a lack of mucosal immunity at the portals of virus entry. The present approach aims to exploit the efficiency of chitosan nanoparticle-encapsulated inactivated type ‘O’ FMDV antigen (FMDV-CS-NPs) to induce mucosal and systemic immune responses in a guinea pig animal model through intranasal and intramuscular administration in comparison with the conventional inactivated, mineral oil-adjuvanted vaccine that is administered systemically. In this study, the FMDV-CS-NPs were prepared by ionotropic gelation, followed by incubation; were characterized for their physical properties and in vitro antigen release; and were found to encapsulate a good amount of antigen. The prepared nanoparticles were assessed for their ability to induce humoral and cell-mediated immune responses by SNTs; indirect ELISAs for serum IgG, IgG1, and IgG2; and nasal washing sIgA and lymphocyte proliferation assays. The preparation induced comparatively more measurable sIgA and systemic immune responses with the intranasal and intramuscular routes of administration, respectively, which are attributable to a specific interaction between the positively charged chitosan and the negatively charged mucosal surface and cell membrane. The challenge infection protected 87.5% of the animals in the FMDV-CS-NP I/M group, followed by 77.7% in the FMDV-CS-NP I/N and inactivated vaccine groups. The outcomes of this study in guinea pigs highlight that chitosan nanoparticle-based vaccine formulations could be employed as a promising antigen delivery system for targeted delivery, devoid of any adverse effect, to induce protective immune responses.

## Simple Summary

The efficacy of nanoparticle-delivered inactivated FMDV in inducing mucosal and systemic immune responses was studied in a guinea pig model. The chitosan nanoparticles encapsulating inactivated FMDV antigen (FMDV-CS-NPs) were prepared by the incubation method and their properties characterized. The immune response and protective efficiency of the FMDV-CS-NPs administered through intranasal and intramuscular routes were evaluated. The FMDV-CS-NPs had a good morphology and high stability, with a mean diameter of 615.3 nm and an encapsulation rate of 64–69%, and a zeta potential of +46.07 mV was produced. The results of the in vitro assay of virus release from the FMDV-CS-NPs indicated that FMDV was released with an initial burst release of 21%. The immune response studies in guinea pigs revealed significantly notable levels of nasal secretory IgA in the intranasally FMDV-CS-NP-immunized group compared with the other groups. A degree of protection as good as in the conventional vaccine group (77.7%) could be observed in FMDV-CS-NP I/N group guinea pigs nonetheless, and it was lower than the highest level of protection (87.5%) observed in the FMDV-CS-NP I/M group. The findings of the study in the guinea pig model highlight that chitosan nanoparticle-based vaccine formulations could be prospectively considered for delivering the antigen to the targeted sites that were devoid of any adverse effect to induce a protective immune response.

## 1. Introduction

Foot and mouth disease (FMD) is a severe, highly contagious viral disease of livestock that has a significant economic impact affecting cattle, swine, sheep, goats, and other cloven-hoofed ruminants [1]. The disease is caused by *Aphthovirus* of the family *Picornaviridae*, and there are seven strains/serotypes (O, A, C, Asia1, SAT1, SAT2, and SAT3), which are endemic in different countries worldwide. Out of the seven serotypes, O, A, and Asia-1 are prevalent in India [2], with an estimated economic loss up to 200 billion INR per annum [3]. Vaccination has a critical role in the effective control of FMD in endemic areas [4]. Inevitably, each serotype requires a specific vaccine to provide immunity to a vaccinated animal, since there is no cross-protection among the serotypes [5]. The properties of the virus to infect and multiply with extraordinary rapidity are key factors that are addressed by veterinarians and researchers to control FMD outbreaks, since the immune system needs a minimum of 5 to 7 days to respond to a vaccine. The severity of FMD can be reduced if serotype-specific antibodies (neutralizing) are available at the right time in sufficient quantities at the right place (portals of infection), to clear or neutralize the invading virus before establishing viremia.

The widely used vaccine contains a chemically inactivated whole virus with an adjuvant to provide protection. Despite the fact that inactivated vaccines are safe, it has its own limitations, like a short duration of immunity [6]; a lack of immunity at the portals of infection [7,8] and a lack of cross-protection against different strains, even within a serotype [9,10]; and a lack of sterile immunity and poor immunogenicity, as the vaccine potency of the 12S particles formed, due to the non-maintenance of the cold chain and variations in the purification procedures, is 45–400-fold less than that of the intact 146S virion commonly present in conventional vaccines [11]. As these inactivated vaccines do not prevent primary infection and protect only from generalization, with a likelihood that more than half of the vaccinated animals will become carriers [12,13], the most effective way to induce mucosal immunity (secretory IgA) is to administer a vaccine directly to the mucosal surface. There are specialized cells in mucosal surfaces that are capable of antigen uptake and of presenting the antigen to professional antigen-presenting cells (APCs), which can stimulate both local and systemic immune responses [14]. Hence, there is a requisite and scope for development of novel strategies to induce effective immunity in mucosal regions besides systemic sites.

Antigens delivered to the mucosal surfaces are subjected to rapid elimination or inactivation by mucosal enzymes and bacterial flora, necessitating a delivery vehicle. Nanoparticle-based antigen delivery has gained impetus due to its biodegradeable, mucoadhesive, immunostimulating, and antimicrobial properties and has been employed to increase the efficacy of vaccines [15]. Antigens encapsulated in polymeric nanoparticles induce antigen-specific cellular and humoral immune responses via specialized delivery of the antigen to the target. Chitosan is a polysaccharide comprising copolymers of glucosamine and N-acetylglucosamine and can be derived by the partial de-acetylation of chitin, a material that is found in abundance in shells of crustacea such as lobsters, prawns, and crabs [16] and that has attracted attention due to its pH sensitivity, biocompatibility, and bioactive functions [17]. Chitosan nanoparticles (CS-NPs) are prepared by different methods, like emulsification, precipitation, ionic or covalent crosslinking, or combinations considering diverse factors, such as size, stability, drug loading capacity, and retention time. Commonly, vaccine antigen-encapsulating CS-NPs are prepared by an ionic gelation method using sodium tripolyphosphate (TPP) as a precipitating agent [18,19]. CS-NPs have been considered a novel vaccine delivery vehicle and a potential mucosal adjuvant [20]. Mucoadhesive chitosan nanoparticles tightly attach to the mucosa and enhance the viscosity of mucin, which leads to the improvement in mucosal absorption [21]. Intranasal administration of chitosan-based norovirus (Norwalk VLP) vaccines demonstrated that chitosan-based vaccines can be effective in preventing disease in humans [22,23]. An inhalable nanovaccine comprising chitosan and SARS-CoV-2 spike protein enabled a strong spike-specific antibody immune response and augmented local mucosal immunity in bronchoalveolar lavage and lungs with potential protection against SARS-CoV-2 [24]. M cells present under the nasal epithelium covering the nasal-associated lymphoid tissue (NALT) are usually responsible for the uptake and delivery of antigens through adsorptive endocytosis to the submucosal lymphoid tissues [25] and induce humoral and cellular immune responses through lymphoid follicles (mostly B cells), dendritic cells, macrophages, and intrafollicular areas (mostly T cells) [26].

In the present study, FMDV-CS-NPs were prepared by ionotropic gelation and characterized for their physical properties and in vitro antigen release. The humoral, mucosal, and cell-mediated immune responses to the FMDV-CS-NPs were assessed by intranasal and intramuscular administration in comparison with the inactivated oil-adjuvanted vaccine in guinea pigs, and the protective efficacy was evaluated by challenge studies.

## 2. Materials and Methods

### 2.1. Virus and Cell Types

Foot and mouth disease virus (FMDV) vaccine strain O IND R2/75 maintained at the FMD Research Centre, Indian Veterinary Research Institute (IVRI), Bangalore, was propagated and used for preparation of viral antigen and immunization, serum neutralization test (SNT), ELISA, and lymphocyte transformation assay. Baby Hamster kidney (BHK)-21 clone 13 (Glasgow) cell line monolayers maintained at the FMD Research Centre, IVRI, Bangalore, were used for virus propagation and SNT.

### 2.2. Laboratory Animals

Healthy albino guinea pigs (*Cavea* sp.) of both sexes, weighing 450–500 g, used in the experiment were obtained from Animal Experimental Station, IVRI, Yelahanka, Bangalore. The guinea pigs were housed in cages and provided ad libitum feed and water. The guidelines of the institutional animal ethics committee were followed for the maintenance, handling, and care of animals. All the animal protocols were reviewed and approved by the Committee for the Purpose of Control and Supervision of Experiments on Animals, Ministry of Environment and Forests, Government of India (#460/01/ab/CPCSEA).

### 2.3. Preparation and Purification of FMDV 146S Antigen

The BHK-21 Cl-13 cell monolayers grown with DMEM supplemented with 2% fetal bovine serum and 1% antibiotic–antimycotic solution comprising 10,000 units/mL of penicillin, 10,000 µg/mL of streptomycin, and 25 µg/mL of Amphotericin B in 6 Povitzky flasks with a volume of 2400 mL at 37 °C were infected with 2% MP-5 of type ‘O’ FMDV. The virus was harvested from the infected BHK cells after 16–18 h of incubation at 37 °C upon the appearance of cytopathic effects (CPEs). The virus suspension was treated with 1% (*v*/*v*) chloroform for 1 h at 4 °C and clarified by centrifugation at 8000 rpm for 30 m at 4 °C in a refrigerated high-speed centrifuge. The supernatant was collected, and 8% (*w*/*v*) polyethylene glycol (PEG) 6000 was added to precipitate the virus. The PEG-treated virus suspension was then left overnight at 4 °C with continuous stirring in a magnetic stirrer. The virus suspension was then centrifuged at 8000 rpm for 30 m at 4 °C. The pellet was collected and suspended in 65× of original volume in Tris-NaCl buffer at pH 7.4 and clarified to remove insoluble debris by centrifugation at 15,000 rpm for 20 m at 4 °C.

The PEG-concentrated, clarified virus was layered on to 8 mL of CsCl gradient (4 mL of 1.38 g CsCl per mL of double distilled water layered over 4 mL of 1.42 g CsCl per ml of double distilled water) in a 36 mL polyallomer ultracentrifugation tube. Ultracentrifugation was performed in a swinging-bucket rotor (AH-629) OTD 75B Sorvall ultracentrifuge at 27,000 rpm for 4 h at 4 °C. The gradient was fractionated from the bottom of the tube using a long siphon tube. Absorbance of the fractions was measured at wavelengths of 259 nm and 239 nm. The fraction with maximum absorbance at 259 and minimum at 239 nm, giving a ratio of 1.3–1.4 at 259/239, were pooled, and 146S concentrations were calculated as an extinction coefficient (E1% 259) of 76 [27]. The fraction containing 146S particles was pooled and dialyzed extensively overnight against Tris-Nacl buffer at pH 7.4 at 4 °C to remove any traces of CsCl salt.

### 2.4. Inactivation of FMDV by Binary Ethyleneimine

The inactivation of virus was performed as per OIE standards [28]. The dialyzed virus was filtered and inactivated by the addition of binary ethyleneimine (BEI). BEI was prepared by dissolving 2-bromoethylamine hydrobromide in a 0.175 N sodium hydroxide solution to a concentration of 0.1 M and incubating it at 37 °C for 1 h under continuous stirring. The BEI thus formed was added to the virus suspension held at 37 °C to give a final concentration of 0.001 M. Inactivation was carried out for 24 h with continuous stirring. After inactivation, residual BEI in the harvest was inactivated by adding 1 M sodium thiosulfate solution to a final concentration of 0.3% (*v*/*v*). The inactivation of virus was confirmed by inoculating in BHK confluent monolayers.

### 2.5. Preparation of Chitosan Nanoparticles (CS-NPs)

The CS-NPs were prepared by an ionic gelation method [29]. Briefly, 0.5% chitosan (>75% deacylated) was prepared in a 1% acetic acid solution (*v*/*v*), and 0.1% sodium tripolyphosphate (TPP) was prepared in distilled water. Consequently 9 mL of TPP was added dropwise to 18 mL of chitosan solution under magnetic stirring at room temperature for 15 min for ionic complexation. The mixture was centrifuged at 9500 rpm for 15 min at room temperature, the supernatant was discarded, and the pellet was redispersed in distilled water and washed twice. The yield of chitosan nanoparticles was calculated as the weight percentage of the final product after drying, with respect to the initial amount of chitosan salt used for the preparation. The prepared nanoparticles were analyzed for size and zeta potential and stored at −20 °C until use.

### 2.6. Preparation of Chitosan Nanoparticles with Inactivated FMDV 146S Antigen

The chitosan nanoparticle-encapsulated FMDV antigen was prepared by the incubation method [30,31]. Briefly, 300 µg of inactivated type ‘O’ FMDV 146S antigen was added to 500 µg of nanoparticles in phosphate-buffered saline and incubated at 37 °C in a shaking incubator at 250 rpm for overnight. The unbound antigen in the supernatant was removed by centrifugation at 3000 rpm for 10 m.

### 2.7. Characterization of Chitosan Nanoparticles

The size, morphology, and the zeta potential of the CS-NPs and FMDV-CS-NPs were examined with Scanning Electron Microscopy (SEM) at the Jawaharlal Nehru Centre for Advances in Scientific Research (JNCASR), Bangalore, India. The nanoparticles were smeared onto a specimen mount, allowed to dry for 2 h, and examined to visualize the surface morphology. The particle sizes of the chitosan nanoparticles and chitosan nanoparticle-encapsulated virus were measured after suspending them in distilled water using the laser diffraction method using Malvern Mastersizer 2000 (Malvern Instruments, Worcestershire, UK). The mean size of the prepared particles in three different batches were considered for the average particle size and expressed as the median diameter. The zeta potential of the nanoparticles was analyzed in Zetasizer Nano Series, NanoZS (Malvern Instruments, Worcestershire, UK).

### 2.8. Estimation of Antigen Loading Efficiency and In Vitro Antigen Release

The loading efficiency (LE) of the antigen into the chitosan nanoparticles was calculated by measuring the difference in the total amount of type ‘O’FMDV antigen added to the chitosan nanoparticles and the amount remaining in the supernatant obtained by centrifugation of antigen-loaded nanoparticle suspension at 3000 rpm for 10 min after incubation at 37 °C under shaking at 250 rpm overnight [32].

$$\mathrm{LE}\;(\%)=\frac{Total\;amount\;of\;FMDV\;antigen\;loaded-Unbound\;FMDV\;antigen}{Total\;amount\;of\;FMDV\;antigen\;loaded}\times 100$$

The release of type ‘O’ FMDV antigen from the FMDV-CS-NPs under in vitro conditions was determined spectrophotometrically. The antigen-loaded chitosan nanoparticles (120 µg) were suspended in phosphate-buffered saline (PBS; pH 7.4) in 1.5 mL centrifuge tubes and kept in a shaking incubator at 100 rpm at 37 °C to facilitate antigen release. At scheduled times, samples were centrifuged at 10,000 rpm for 15 min, and the supernatant was replaced with fresh PBS. The amount of FMDV antigen released from the FMDV-CS-NPs in the supernatant collected at different time intervals was determined by subtracting the total amount of antigen that was loaded into the nanoparticles.

### 2.9. In Vivo Immunization Study

The guinea pigs were allotted to 6 groups, each having 10 animals and being administered with different antigen formulations, boosted on day 21, as shown in Table 1.

### 2.10. Collection of Serum and Nasal Washing

Serum samples were collected 0 (pre-immunization), 7, 14, 21, and 28 days post immunization (dpi) from the experimentally immunized guinea pigs, heat-inactivated at 56 °C for 30 min, and stored at −20 °C until further use. Nasal washings were collected by instilling 400 µL of sterile PBS at pH 7.4 (200 µL in each nostril) into the nostrils by using an air displacement pipette 0 (pre-immune), 7, 14, 21, and 28 dpi. The nasal flush was then collected and briefly centrifuged for clarification. The supernatant was collected carefully, treated with the protease inhibitor phenylmethylsulfonyl fluoride (PMSF; 1 mM), and stored at −20 °C for further use.

### 2.11. Serum Neutralization Test

Sera collected from guinea pigs 0 (pre-immune), 7, 14, 21, and 28 dpi were tested for virus neutralizing activity by a microserum neutralization test using BHK-21 cells in microtiter plates as per the standard procedure [4]. A volume of 50 µL of two-fold dilution of sera in sextuplicate from 1:2 to 1:256 in Eagle’s maintenance medium was mixed with 50 µL per well of 100 TCID_50_ FMDV ‘O’ suspension, and the plates were incubated at 37 °C for 1 h. Then, 50 µL of BHK-21 cells (2 × 10^4^ cells/mL) was added to each well and further incubated in a humidified chamber containing 5% CO_2_ at 37 °C for 48 h. Every assay had appropriate serum, virus, and cell controls. The plate was observed with an inverted microscope after 48 h of incubation to record changes like the rounding and detachment of the cells, and the neutralizing antibody titre was determined using the Reed–Muench method. The neutralizing antibody titres (NATs) were calculated as the log10 of the reciprocal of the final serum dilution that neutralized 100 TCID_50_ of virus in 50% of the wells.

### 2.12. Quantification of Secretory IgA (sIgA) in Nasal Washing

The type ‘O’ FMDV-specific mucosal immune response in terms of IgA antibody levels in nasal washing samples was assessed by an indirect ELISA. Briefly, 96-well polystyrene microtiter plates (NUNC maxisorp) were coated with 10 µg/mL of purified inactivated type ‘O’ 146S FMDV antigen (100 µL/well) overnight at 4 °C in coating buffer (0.05 M, pH 9.6). The plates were washed thrice with PBS containing 0.05% Tween-20 (PBS-T). The wells were blocked with 200 µL of 1% BSA in PBS-T for 1 h at 37 °C and then washed thrice as above. The nasal washing sample was then added as a 1:1 dilution in duplicate wells @ 50 µL/well and incubated for 2 h at 37 °C. The plates were washed thrice again. Then, a 1:400 dilution of rabbit anti-guinea pig IgA (alpha-chain specific; Bethyl Laboratories, Montgomery, TX, USA) in 100 µL/well was added to the wells and incubated at 37 °C for 1 h. The plates were then washed thrice, and 100 µL of a 1:10,000 dilution of secondary antibody (HRPO-conjugated goat anti-rabbit IgG) (Sigma-Aldrich, Bengaluru, India) was added to the wells. The plates were incubated at 37 °C for 1 h and washed thrice. After the final washing, 50 µL of citrate buffer at pH 5.0 with 4 mg of O-phenyldiamine hydrochloride (OPD) and 6 µL of H_2_O_2_/6 mL was added to the wells, and the plate was incubated at 37 °C for 15 m in the dark. The reaction was stopped by adding 1 M H_2_SO_4_, and absorbance was measured at 492 nm in a ELISA reader (BioRad, Bengaluru, India).

### 2.13. Quantification of Serum Antibodies by Indirect ELISA

The IgG and its subtypes, viz., IgG1 and IgG2, in serum samples were estimated by an indirect ELISA to assess the ability of the nanoparticle-delivered antigen to induce a humoral immune response. Briefly, polystyrene 96-well microtiter plates were coated overnight with 10 µg/mL purified inactivated virus (100 µL/well) in coating buffer at 4 °C. The plates were washed thrice with PBS-Tween 20 (0.05%) and blocked with 200 µL/well of 1% BSA in PBS-T for 1 h. Then, the plates were thoroughly washed thrice, and 100 µL of 1:100 [33] and 1:50 [34] dilutions of respective serum samples in PBST solution was added to each well and incubated at 37 °C for 1 h. Following washing thrice with PBST, 100 µL of 1:1000 dilutions of peroxidase-conjugated goat anti-guinea pig IgG (Sigma, Bengaluru, India) and 1:5000 goat anti-guinea pig IgG1 and IgG2 (ICL) in 1% BSA/PBS-T was added to the wells and incubated at 37 °C for another 1 h. Subsequently, plates were washed thrice as described earlier, and 50 µL/well of substrate buffer with 4 mg of O-phenyldiamine hydrochloride (OPD) and 6 µL of H_2_O_2_/6 mL was added to the wells; the plate was incubated at 37 °C for 15 m in the dark for development of color, the reaction was stopped by adding 1 M H_2_SO_4_, and absorbance was measured at 492 nm with a BioRad ELISA reader.

### 2.14. Lymphocyte Transformation Assay (LTA)

Peripheral blood mononuclear cells from the immunized guinea pigs were isolated by density gradient centrifugation [35]. Briefly, 3 mL of diluted heparinized blood (1:1 in PBS) was overlaid on 1.5 mL of Histopaque 1083 in a 15 mL conical centrifuge tube and centrifuged at 1700 rpm for 30 min at room temperature. The cells at the interface containing mononuclear cells were carefully collected in RPMI-1640 medium with a micropipette and washed twice by centrifugation at 2000 rpm for 10 m. The cells were finally washed with RPMI-1640 medium containing 10% fetal calf serum (FCS). The cell pellet was reconstituted in RPMI1640, and the cell count and viability were ascertained by the trypan blue dye exclusion method.

A lymphoproliferation assay was performed using MTT (3-(4, 5-dimethylthiazol-2-yl)-2, 5 diphenyl tetrazolium bromide) dye [36]. Briefly, 100 µL of PBMCs (1 × 10^6^ cells/mL) in RPMI-1640 medium containing 10% FCS and antibiotics (50 µg/mL streptomycin, 100 IU/mL penicillin, and 50 µg/mL gentamicin) was dispensed to 96-well cell culture plates, and the final volume of the respective wells were made to 200 µL with medium containing antigen (10 µg per well), medium alone (control unstimulated wells), or concanavalin A (10 µg/mL) as a positive control for in vitro proliferation assay. The plates were incubated at 37 °C for 72–96 h in a humidified atmosphere having 5% CO_2_. After incubation, 10 µL of MTT (5 mg per ml) solution prepared in sterile PBS was added to each well, and the plates were further incubated at 37 °C in 5% CO_2_ for 4 h. The plates were centrifuged at 1500 rpm, and 100 µL of the supernatant was removed and replaced with 100 µL of dimethyl sulfoxide (DMSO) and incubated at 37 °C for 15 min in the dark to dissolve the formazan crystals formed by metabolization of MTT. The absorbance of the plate was measured at 550 nm in an ELISA reader. The stimulation index was calculated as detailed below:

$$\mathrm{Stimulation}\;\mathrm{Index}\;(\mathrm{SI})\;=\;\frac{OD\;of\;stimulated\;culture}{OD\;of\;unstimulated\;culture}$$

### 2.15. Challenge Study

The protective efficacy of the preparations was assessed by challenging all the immunized groups with 0.2 mL of 10^3^ GPID_50_ of homotypic virulent serotype ‘O’ FMDV by footpad tunnelling in the left rear leg after 28 days of immunization and monitoring for 8 consecutive days for the development of characteristic FMD lesions on the remaining footpads. On day 8 post-challenge, the severity of lesions was scored as protected for the animals showing primary lesions at the site of virus inoculation without spreading to the other feet and as unprotected if there were secondary lesions in the remaining footpads other than the inoculated footpad due to systemic viremia [37].

### 2.16. Statistical Analysis

The results of the ELISAs, log_10_ SNTs, and lymphocyte proliferation assays were expressed as means ± standard errors. The data were analyzed with the help of Graphpad Prism 5.0 software. Bonferroni post n tests and two-way analysis of variance were used to test the statistical significance, and *p*-values less than 0.05 were considered statistically significant.

## 3. Results

### 3.1. Propagation of FMD Virus and Preparation of 146S FMDV Antigen

The large-scale production of virus for the immunization and challenge studies was effectively achieved by infecting BHK-21 cell monolayers cultured in Povitzky flasks, and CPEs such as cell rounding and complete detachment were observed after 16 h of infection. The density gradient centrifugation of PEG-concentrated type ‘O’ virus using cesium chloride gradient yielded pure and highly concentrated virus in the second-to-fourth fractions (Table 2). All the fractions having an A259/A239 ratio of 1.3–1.4 were pooled, dialyzed, and quantified for further applications.

### 3.2. Confirmation of FMD Virus Inactivation and Quantification of Antigen

The pooled and concentrated 146S virus fractions inactivated with BEI and treated with sodium thiosulphate did not produce any CPE on BHK-21 monolayer cells upon inoculation. The total concentration of 146S FMDV ‘O’ antigen after inactivation in the pooled fractions was found to be 200 µg/mL. The purified, inactivated, dialyzed, and filtered type ‘O’ FMDV was used in vaccine preparation and immunological assays like LTA and ELISA.

### 3.3. Characterization of Nanoparticle Morphology

**Figure 1:**
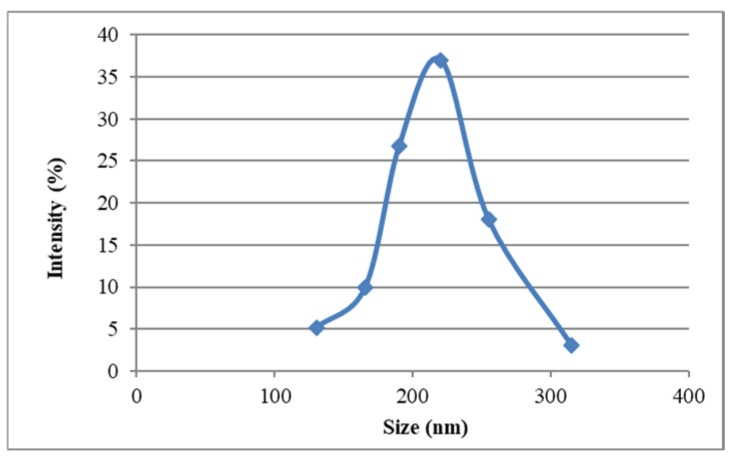
Size distribution of chitosan nanoparticles. The blue dots are indicative of the number of nanoparticles present in a particular size range.

**Figure 2:**
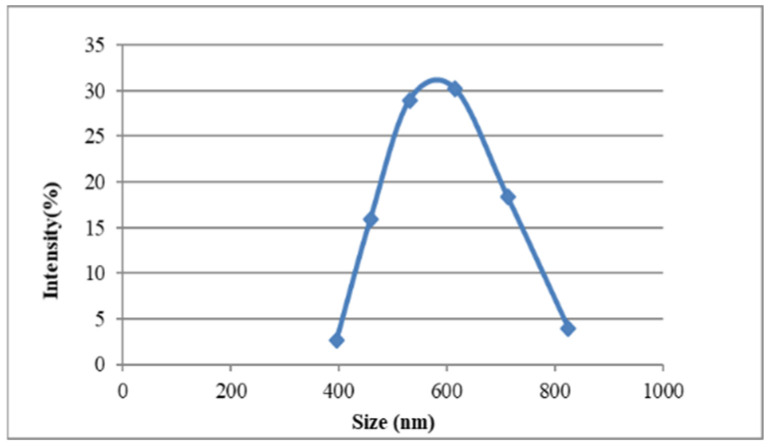
Size distribution of FMDV-CS-NPs. The blue dots are indicative of the number of nanoparticles present in a particular size range.

**Figure 3:**
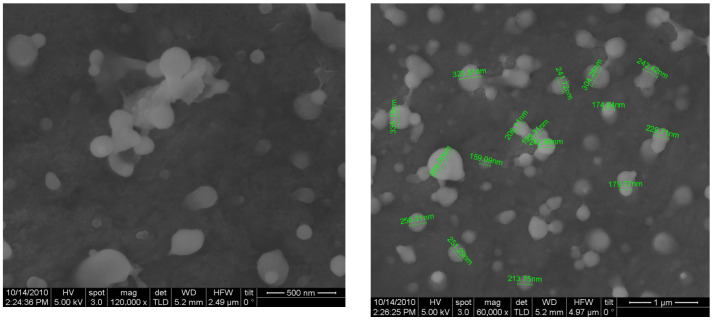
Scanning Electron Micrograph of chitosan nanoparticles.

**Figure 4:**
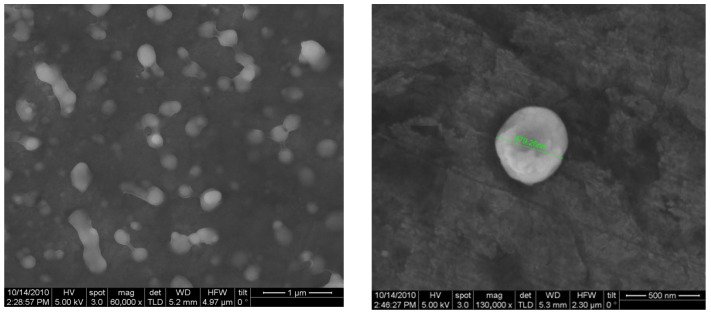
Scanning Electron Micrograph of FMDV-CS-NPs.

**Figure 5:**
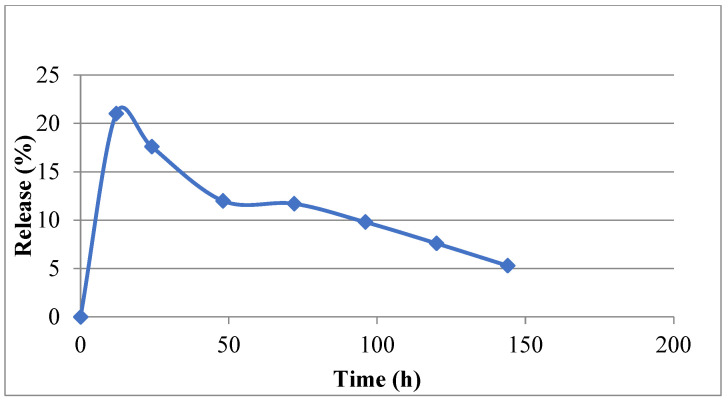
In vitro release of type ‘O’ FMD virus from chitosan nanoparticles. The blue dots are indicative of the number of virus particles released from the encapsulated nanoparticles at different time points.

The chitosan nanoparticles formed instantly upon addition of polyanionic TPP to the chitosan solutions. The mean particle size of the prepared chitosan nanoparticles was found to be 220 nm (37%), and the polydispersity index (PDI) was found to be 0.301. The size distribution of the chitosan nanoparticles ranged from 130.21 to 315.27 nm (Figure 1). The average zeta potential of the chitosan nanoparticles was around +46.07 mV (Table 3). The mean particle sizes of the type ‘O’ FMDV-encapsulating chitosan nanoparticles were comparatively much larger than the chitosan nanoparticles (615.13 nm), ranging from 396.05 to 824.99 (Figure 2), and the PDI was 0.421. The average zeta potential of the virus-loaded chitosan nanoparticles was +17.64 mV (Table 4). The surface morphology of the CS-NPs in SEM was found to be spherical and smooth, with sizes ranging from 159 to 467 nm, with some of the small chitosan nanoparticles being fused into larger ones (Figure 3). Similarly, the surface morphology of the virus-loaded chitosan was also determined to be spherical, with sizes ranging from 396 to 824 nm, and agglomeration of the virus-loaded chitosan nanoparticles could also be observed (Figure 4). The average loading efficiency of the virus onto the nanoparticles was observed to be 66.8 ± 2.3%, ranging from 64.2 to 68.6% (Table 5). The release of FMDV antigen from the chitosan nanoparticles in PBS at 37 °C showed a burst release of 21% in the initial 8 h, followed by slow and sustained release till the 150 h mark (Figure 5).

### 3.4. Immunization and Evaluation of Immune Responses Against Vaccine Preparations

The guinea pigs immunized with nanoparticle preparations through intranasal or intramuscular routes were observed for a period of 28 days; no untoward reaction could be noticed, and they were apparently healthy.

#### 3.4.1. Serum Neutralization Assay to Estimate the Neutralizing Antibodies

**Figure 6:**
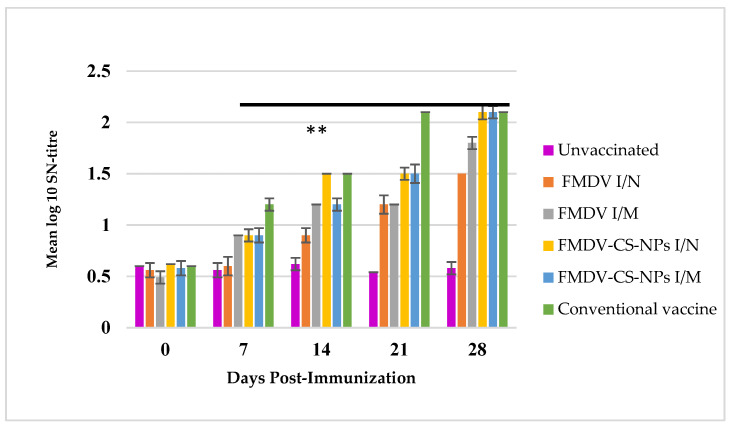
FMDV-specific serum neutralizing antibody titre (log_10_ SN) in different immunized groups with a significant increase in NATs from day 7 onwards, reaching the maximum in the FMDV-CS-NP I/M and I/N groups, comparable to the conventional vaccine group on day 28 after the administration of a booster dose on day 21. The black line with ** indicates significance at *p* < 0.01 between the groups and days.

The virus neutralizing antibodies in the serum expressed in mean log10 SN per ml could be detected as early as 7 dpi. On day 0, there was no notable difference in NATs among the experimental groups, while the NATs showed an increasing trend in all immunized groups from day 7 and remained static from day 14 to 21. The NATs were less than 1.5 in all the groups till 21 dpi, except for FMDV-CS-NP I/M and I/N, which showed 1.5, and the conventional vaccine-immunized group, with the highest NAT of 2.1. Surprisingly, a significant (*p* < 0.01) rise in SN titre reaching 2.1 in FMDV-CS-NP-immunized groups (I/M and I/N) comparable to the conventional vaccine group was observed on day 28 after the administration of a booster on day 21 (Figure 6).

#### 3.4.2. Nasal Secretory IgA Response Against Vaccine Preparations

**Figure 7:**
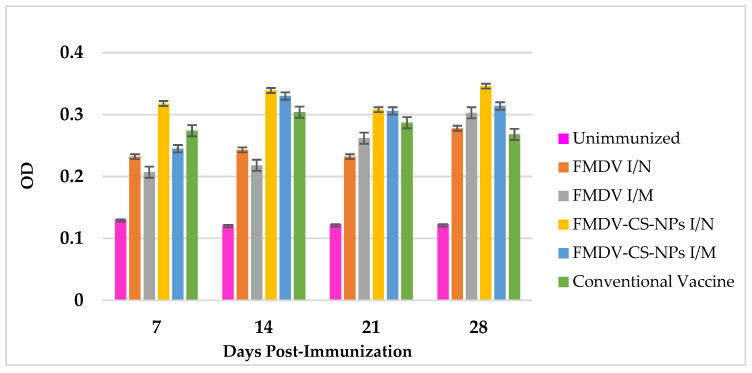
FMDV-specific nasal sIgA antibody responses in immunized guinea pigs showing a noticeable difference in all the immunized groups, including the conventional vaccine group, on day 7 with the highest sIgA levels in the FMDV-CS-NP I/N group on days 7 and 14, followed by a less marked improvement in sIgA levels even after administration of a booster dose.

The secretory IgA levels in the nasal washing of the immunized groups could be observed from 7 dpi onwards. Interestingly, both routes of immunization evoked IgA responses; however, the intranasal virus-loaded chitosan group elicited the quickest and highest response. The earliest and highest IgA antibody levels were found in the FMDV-CS-NP I/N group on days 7 (0.318 ± 0.004) and 14 (0.339 ± 0.002); however, the IgA levels decreased on day 21. The second highest IgA levels were found in FMDV-CS-NP I/M group (0.330 ± 0.008) on day 14. Similar to the SNT response, a significant (*p* < 0.01) increase in IgA levels of all the immunized groups could be observed on day 28 upon administration of a booster on day 21. Notably, the highest IgA titre was seen in the FMDV-CS-NP I/N group (0.346 ± 0.007) on day 28. Unsurprisingly, the conventional vaccine elicited the lowest IgA titre on day 28 even after booster dose administration among the immunized groups (Figure 7).

#### 3.4.3. Total IgG Response Against Vaccine Preparations

**Figure 8:**
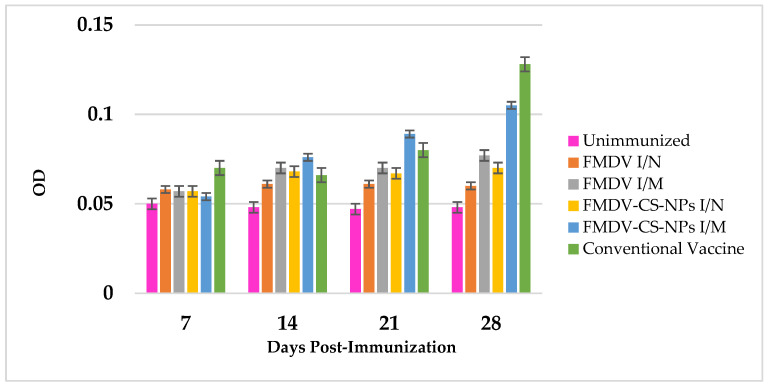
FMDV-specific total IgG response in immunized guinea pigs indicating a significant antigen-specific total IgG response in the groups (*p* < 0.05) on different days from 7 dpi to 21 dpi in all the groups, with a highly significant surge in the conventional vaccine group, followed by the FMDV-CS-NP I/M group on day 28.

A significant antigen-specific total IgG response could be observed in the groups (*p* < 0.05) on different days, with an increasing trend from day 7 to day 21 in all the groups and a substantial surge in the conventional vaccine group (0.128 ± 0.04) (*p* < 0.01), followed by the FMDV-CS-NP I/M group (0.105 ± 0.01) on day 28 after the administration of a booster on day 21, and only a little difference in the other immunized groups (Figure 8).

#### 3.4.4. IgG1 Response Against Vaccine Preparations

**Figure 9:**
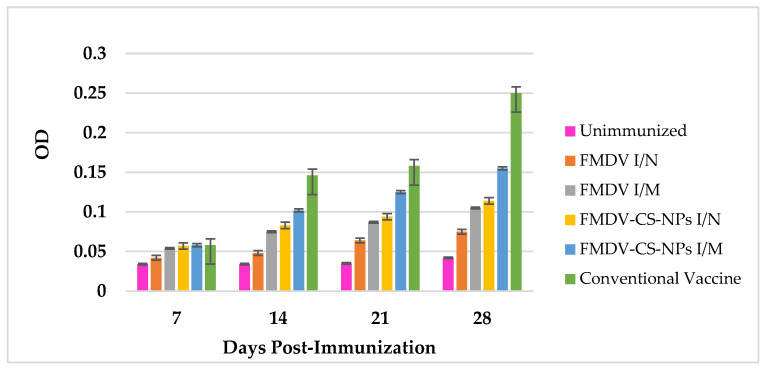
FMDV-specific IgG1 antibody response in immunized guinea pigs indicating a significant IgG1 response only in FMDV-CS-NP I/M and a highly significant response in the conventional vaccine group 14 dpi and 28 dpi following booster administration.

The type ‘O’ FMDV-specific IgG1 response was found to be significant in the groups (*p* < 0.05), and a meagre difference was observed on day 7. However, the IgG1 level started increasing from day 14 in all the immunized groups, with the conventional vaccine group showing significantly (*p* < 0.01) the highest level (0.250 ± 0.006), followed by the FMDV-CS-NP I/M group (0.155 ± 0.002) and the FMDV-CS-NP I/N group (0.114 ± 0.004) on day 28 (Figure 9).

#### 3.4.5. IgG2 Response Against Vaccine Preparations

**Figure 10:**
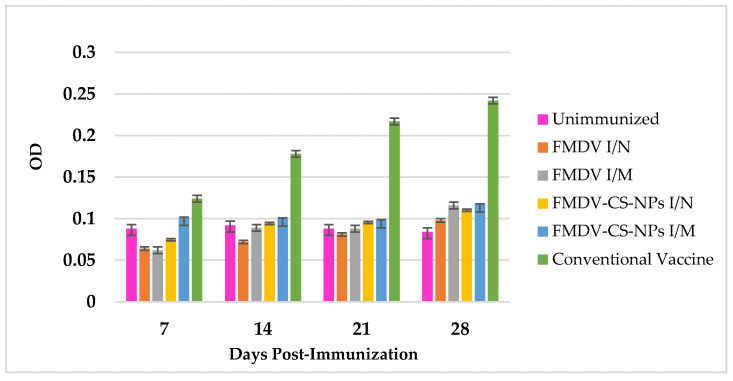
FMDV-specific IgG2 antibody response in immunized guinea pigs highlighting a less pronounced IgG2 response in the groups on different days post-immunization, with only the conventional vaccine group eliciting a remarkable response throughout the immunization study.

The type ‘O’ FMDV-specific IgG2 response was found to be significant in the groups (*p* < 0.05), exhibited an increasing trend from 7 to 14 dpi and remained static till day 21 in all the immunized groups, except for the conventional vaccine group, followed by a moderate increase on day 28 after booster dose administration on day 21. The highest IgG2 antibody level was observed in the conventional vaccine group (0.242 ± 0.004) followed by the FMDV-CS-NP I/M group (0.117 ± 0.009) (*p* < 0.05) on day 28. A highly significant difference in the IgG2 antibody levels could be noted 14, 21, and 28 dpi in the conventional vaccine group (Figure 10).

### 3.5. Lymphocyte Transformation Assay to Estimate CMI Response

**Figure 11:**
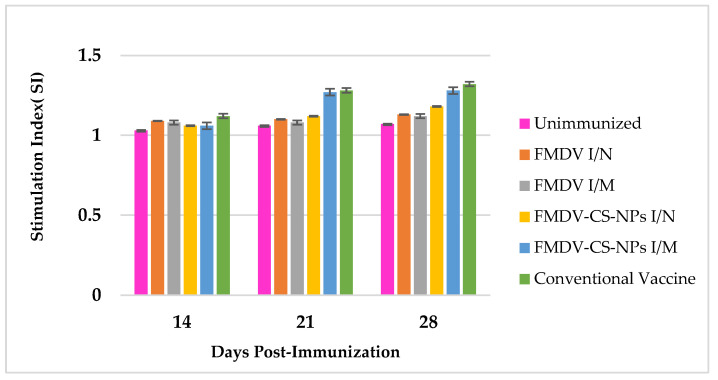
Lymphocyte proliferation response of immunized guinea pigs highlighting a significant change in the stimulation index only 21 dpi, with a maximum response in the conventional vaccine group, followed by the FMDV-CS-NP I/M group.

A significant change in the stimulation index could be evidenced only 21 dpi, while the maximum stimulation index was seen in the conventional vaccine group (1.32 ± 0.016) followed by the FMDV-CS-NP I/M group (1.28 ± 0.021), and a considerable SI (1.18 ± 0.003) was observed in the FMDV-CS-NP I/N group 28 dpi. The type ‘O’ FMDV administered via either the I/N or I/M route without chitosan nanoparticles could not induce marked stimulation indices even after administration of a booster dose on day 28 (Figure 11).

### 3.6. Challenge Infection to Assess the Protection Efficacy of the Vaccine Preparations

**Figure 12:**
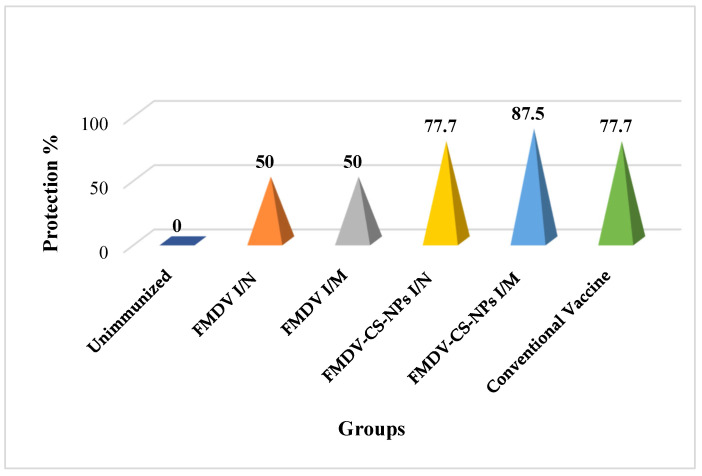
Protection percentage of immunized guinea pigs upon challenge infection denoting the highest level of protection in the FMDV-CS-NP I/M group, followed by the FMDV-CS-NP I/N group and conventional vaccine group.

The FMDV-induced lesions in the immunized guinea pigs scored on day 8 following challenge with 10^3^ GPID_50_ showed no protection in the unvaccinated group, while groups 2 and 3, virus-immunized without nanoparticles through the I/N and I/M routes, showed 50% protection. Interestingly, about 77% of guinea pigs were protected in the FMDV-CS-NP I/N group and conventional vaccine group, whereas the highest level of protection (87%) was seen in the FMDV-CS-NP I/M group (Figure 12).

## 4. Discussion

Foot and mouth disease lingers as a challenging transboundary animal disease with significant economic impact on domestic ruminants, and vaccination with regionally matched serotypes is in vogue as the chief control strategy. India aims to control FMD by 2025 and eradicate it by 2030 through biannual vaccination to induce complete protection. The disease in ruminants is being controlled by administering oil-adjuvanted trivalent (FMDV serotypes O, A, and Asia-I) vaccine containing inactivated 146S antigen to all the cattle and buffalo above 4 months of the age, followed by a booster 1 month after the primary vaccination. The major objective of controlling FMDV infection is to induce the production of neutralizing antibodies, which are detected as early as 4 dpi, peak 14 dpi, and are maintained for very long periods of time (years) [38]. The humoral immune response induced by infection or vaccination protects the animal against FMD but does not consistently prevent replication in the nasopharynx and establishment of persistent infection or carrier status [39]. Hence, the mucosal immune response is highly critical in addition to the systemic immune response not only to combating FMDV infection but also preventing the development of carrier animals. In this context, there is a pressing necessity to induce neutralizing antibodies at the portals of entry of FMDV in the mucosal sites, such as nasopharynx and oral mucosa, particularly the soft palate and palatine tonsils in cattle, critically in the oropharyngeal region, to prevent the attachment of virus to host epithelial cells at the entry point and prevent further systemic spread of infection. The present study was postulated to deliver inactivated FMDV to the mucosal sites by encapsulating it in chitosan nanoparticles, which not only protect the virus from degradation but are also effective in targeted delivery, as they possess inherent mucoadhesive properties due to their positive charge at physiological pH, which allows them to adhere to the negatively charged mucosal surfaces, enhancing the residence time and absorption of the encapsulated drugs [40].

FMDV propagation in BHK-21 cell monolayers yielded a good quantity of virus, as these cell express integrin receptors on their surface for virus attachment, which makes them highly sensitive to replication [41]. Likewise, PEG 6000 effectively precipitated the virus in the cell culture supernatant without affecting its conformation [42]; PEG precipitation is appealing due to its simplicity and the precipitation of virus in low-temperature, high-salt environments, which stabilizes viral particles. The intact FMD virion particles having a sedimentation coefficient of 146S (146S particles) are identified as highly immunogenic components [11]. Since neutralizing antibody production directly correlates with the integrity of 146S antigen [43], the CsCl density gradient ultracentrifugation method yielded fractions with a A259/A239 ratio of 1.3–1.4, containing purified antigen of 200 µg/mL expected to be close to 140S [44]. The inactivation efficacy of 1 mM BEI against FMDV assessed in the BHK-21 monolayer cells ensured the lack of residual live virus in the preparation due to the absence of any observable CPE [4], which re-establishes BEI as an effective inactivating agent against FMDV. Similar findings could be observed in a study [45] where BEI at 1.6 mM was able to inactivate FMD virus within 8–10 h and increasing the concentration of BEI beyond 1.6 mM did not appreciably improve the inactivation process.

The ionic gelation or crosslinking method used to prepare chitosan nanoparticles (CS-NPs) yielded smooth, evenly distributed spherical particles in the range of 130.21 to 315.27 nm, with an average size of 220 nm (37%). The findings of this study align with the results previously reported in ref. [46], where CS-NPs of 200–300 nm were prepared by an ionic gelation method using TPP as the crosslinker. Conversely, the particle size of FMDV-CS-NPs was comparatively much greater than that of CS-NPs and was in the range of 396.05 to 824.99 nm, with a mean diameter of 615.13 nm, while a size range of 147.72 to 594.4 nm with an average particle size of 371.1 nm was noted for the NDV-CS-NPs [47]. Similarly, the CS-NPs and FMDV-CS-NPs prepared in the present study had PDIs, a measure of the uniformity of particle sizes within a sample, of 0.301 and 0.421, respectively [48]. The suitability of nanocarrier formulations for a particular route of drug administration depends on their average diameter, PDI, and size stability, among other parameters [49]. The average zeta potential of the CS-NPs and FMDV-CS-NPs were around +46.07 mV and +17.64 mV, respectively. Similar findings were reported for NDV-CS-NPs with +2.84 mV [47], while +44.1 mV was reported for chitosan and venom-loaded chitosan nanoparticles [48]. The average loading efficiency of the virus onto the nanoparticles (FMDV-CS-NPs), a crucial parameter to assess the suitability of a delivery system, was observed as 66.8 ± 2.3 per cent, ranging from 64.2 to 68.6 per cent, which could be attributed to the size, charge, and surface properties of the virus and its electrostatic interaction with chitosan. The release of FMDV antigen from the FMDV-CS-NPs in PBS at 37 °C showed a burst release of 21 per cent in the initial 8 h, followed by a slow and sustained release till the 150 h. This is comparable to an initial burst release of about 60 per cent in the first 10 h [48], which was attributed to the dissociation of protein molecules that were loosely bound to the surface of chitosan nanoparticles [50] and to the effect of diffusion of protein molecules dispersing close to the surface of nanoparticles [51], followed by a slow release of 30 per cent for the subsequent 62 h, which was related to the slow release of entrapped protein molecules at an approximately constant rate arising from the slow degradation of nanoparticles [52], from the venom-loaded chitosan nanoparticles.

Neutralizing antibodies are crucial to protection against FMDV infection, and the titre is often assessed by serum neutralization tests (SNTs). The significant difference in the NAT FMDV-CS-NP I/N and I/M groups 21 dpi, though lesser than the conventional group, and a comparable NAT 28 dpi after the booster represent evident proof of the potency of the nanoparticle-delivered antigens to elicit a noticeable humoral immune response in the guinea pig, the pertinent animal model, which could be attributed to the protection of FMDV antigen from degradation, as well as sustained release of antigen from the CS-NPs. Identical observations were reported in ferritin-based nanoparticles, which displayed a neutralizing epitope of FMDV in guinea pigs with a notable NAT on day 21 and a significant increase on day 35 upon booster administration [53].

The presence of secretory IgA (sIgA) at the portals of entry of the virus, essentially the respiratory tract, is highly critical to averting the establishment of FMDV infection and further spread within the animal. In this study, a considerable rise in the sIgA levels in the nasal washings of all the immunized groups could be observed as early as 7 dpi onwards, with the FMDV-CS-NP I/N group showing the highest and earliest titres, which were maintained till 14 dpi, followed by a decline 21 dpi and a resurge on day 28 after the booster on day 21. The second-highest sIgA titre in the FMDV-CS-NP I/M group on day 14 and a significant (*p* < 0.01) rise in sIgA levels in all the immunized groups on day 28 upon administration of a booster dose certainly demonstrate the ability of the nanoparticle-delivered antigens to induce a better mucosal immune response against the lowest sIgA titre evoked by the conventional vaccine even after booster dose administration; this is because memory B cells preferentially localize at antigen entry sites, as well as by rapid plasmacytic differentiation, thereby capturing antigens faster than unprimed naïve B cells in B-cell follicles [54]. These findings are supported by a report stating that NDV-CS-NPs induced a better mucosal immune response compared with the LaSota live vaccine [47]. Similar findings state that after intranasal infection with an influenza virus, virus-specific memory B cells develop in the lungs and persist for a long time along with germinal center B cells and plasma cells, which appears to be a unique feature of the mucosal memory response [55]. Moreover, it is highlighted that the targeted delivery of antigens through mucoadhesive chitosan nanoparticles through M (microfold) cells initiates a robust antigen-specific mucosal immune response [56]. Interestingly, the presence of a comparatively higher mucosal antibody response in the chitosan-associated groups compared with the other groups suggests the role of mucosal antibodies in conferring protection against footpad challenge infection.

On the whole, the maximum systemic immune response in terms of total IgG, IgG1, and IgG2 antibody levels against FMDV antigen could be observed in the conventional vaccine group, followed by the FMDV-CS-NP I/M and FMDV-CS-NP I/N groups. These observations evidently imply that though chitosan-mediated delivery systems could induce a notable level of systemic humoral immune response, the levels are slightly lower than those in the conventional vaccine group. These findings could be correlated with the initial burst release of antigen from the CS-NPs in the in vitro antigen release study [51] and the unavailability of enough antigens for sustained release to stimulate the immune cells, considered that the prime feature of the mineral oils present in the conventional vaccine is to slowly release the antigen and induce a better immune response. These findings further confirm earlier reports claiming that Montanide ISA 720 formulated as a water-in-oil (*w*/*o*) emulsion provides a ‘depot effect’, a slow-release system that allows the antigen to be presented to the immune system for a longer period by triggering an inflammatory response, further activating the immune system to respond more strongly and substantially induce Th2 (CD4+) and CTL (CD8+) cells to enhance both humoral and cellular immune responses [57,58].

Although it is generally accepted that protective immunity to FMDV is mostly associated with the neutralizing antibody response, cellular immunity is crucial to inducing antibody production, persistence, and viral clearance [59]. Regarding the cell-mediated immune response, no significant lymphocyte proliferation could be evidenced 14 dpi in none of the immunized groups, including the conventional vaccine group. Although a substantial improvement and meager proliferative response in the FMDV-CS-NP I/M and conventional vaccine groups could be observed 21 and 28 dpi, respectively, this signifies that the inactivated antigens are inefficient in eliciting an effective T-cell-mediated immune response comparable to that of live virus. Surprisingly, the challenge study revealed a different scenario, whereby the maximum protection percentage (87.5%) observed in the FMDV-CS-NP I/M group was even greater than that in the conventional vaccine group (77.7%) and the FMDV-CS-NP I/N group, indicating a role of mucosal antibodies in neutralizing viruses at the primary sites of replication, thereby impeding the systemic establishment of infection in animals. Our findings are in support of the opinion stating that animals with low levels or absence of neutralizing antibodies may still be protected when challenged with FMDV [60]. The outcomes of this study in guinea pigs, the relevant animal model to study FMDV, highlights that chitosan nanoparticle-based vaccine formulations could be promising antigen delivery systems for targeted delivery devoid of any adverse effect to induce a protective immune response.

## 5. Conclusions

In conclusion, the preparation of chitosan nanoparticles by an ionotropic gelation method which does not need any organic solvent makes it more attractive and safer compared with the preparations made using polymers requiring utilization of an organic solvent. The prepared nanoparticle formulations had appropriate size, positive surface charge, and high loading efficiency. The in vitro release study showed initial burst release of antigen followed by slow and steady release. The chitosan-encapsulated FMDV antigens elicited a sensible neutralizing and mucosal antibody response but a lower CMI response. The comparatively lower immune response in the guinea pigs immunized with virus-encapsulating chitosan nanoparticles can be attributed to the instability of the chitosan nanoparticles in physiological fluids like PBS due to burst release of antigen, deprotonation, and the nature of the antigen, together with its complex interactions with the nanoparticles, mainly observed as a reduction in the cationic surface charge of the nanoparticles, which could influence the uptake and delivery of the antigen from the nasal barrier. Overall, chitosan-delivered antigen induced a better mucosal immune response with a maximum protection percentage despite lower CMI in comparison with the conventional vaccine.

## Figures and Tables

**Table 1:** Immunization groups and route of administration.

Group	Inoculum	Route of Immunization
I	Unvaccinated control	-
II	Inactivated type ‘O’ FMDV (2 µg/100 µL)	Intranasal (I/N)
III	Inactivated type ‘O’ FMDV (2 µg/100 µL)	Intramuscular (I/M)
IV	Chitosan loaded with inactivated type ‘O’ FMDV (2 µg/100 µL)—FMDV-CS-NPs	Intranasal (I/N)
V	Chitosan loaded with inactivated type ‘O’ FMDV (2 µg/100 µL)—FMDV-CS-NPs	Intramuscular (I/M)
VI	Inactivated mineral oil-adjuvanted type ‘O’ FMDV vaccine (2 µg)	Intramuscular (I/M)

**Table 2:** Quantitation of 146S particles by CsCl density gradient centrifugation.

Fraction	OD at 259 nm	OD at 239 nm	A259/A239
1	0.9546	1.2442	1.3033
2	1.0663	1.4975	1.404
3	1.0078	1.4276	1.416
4	0.9076	1.2969	1.428
5	1.6836	2.0839	1.2377
6	2.8817	3.2520	1.128

**Table 3:** Zeta potential of chitosan nanoparticles.

Count (Number of Particles)	Zeta Potential (mV)
492,148	46.97
472,650	50.09
402,467	53.21
267,946	56.34
153,962	40.72
348,875	43.85
29,759	31.36

**Table 4:** Zeta potential of FMDV-CS-NPs.

Count (No. of Particles)	Zeta Potential (mV)
19,266	23.89
42,073	20.77
230,090	11.4
200,554	17.64
319,763	14.52

**Table 5:** Loading efficiency of chitosan nanoparticles with type ‘O’ FMD virus.

Sample	Total Amount of Antigen Loaded (µg)	Unbound Antigen in Supernatant (µg)	Loading Efficiency (%)
1	280	100	64.2
2	500	157	68.6
3	350	113	67.7
Average loading efficiency = 66.8 ± 2.3			

## Data Availability

The original contributions presented in this study are included in the article. Further inquiries can be directed to the corresponding authors.
